# High-affinity transferrin receptor binding improves brain delivery of bispecific antibodies at tracer dose

**DOI:** 10.1186/s12987-025-00693-2

**Published:** 2025-08-21

**Authors:** Gillian Bonvicini, Sunitha Singh, Lisa Sandersjöö, Tiffany Dallas, Eva Schlein, Amelia D. Dahlén, Sara Lopes van den Broek, Dag Sehlin, Ken G. Andersson, Stina Syvänen

**Affiliations:** 1 Department of Public Health and Caring Sciences, Uppsala University, Uppsala, Sweden; 2 BioArctic AB, Stockholm, Sweden

**Keywords:** Affinity, Alzheimer’s disease, Amyloid-β, Positron emission tomography (PET), Transferrin receptor (TfR)-mediated transcytosis

## Abstract

**Background:**

Transferrin receptor (TfR)-mediated transcytosis is a well-established method for delivering biologic therapeutics and diagnostics to the brain. Although moderate affinity towards TfR is beneficial for TfR-mediated brain delivery at therapeutic doses, emerging evidence has indicated that high TfR affinity may be more beneficial at tracer doses. With the development of antibody-based PET radioligands for neurodegenerative diseases, such as Alzheimer’s disease, understanding the pharmacokinetics of TfR-binders at tracer dose is essential. Thus, this study aimed to evaluate the effect of TfR affinity on brain uptake at a tracer dose in both wild-type (WT) and amyloid-beta (Aβ) pathology presenting mice and to demonstrate the usability of TfR-mediated brain delivery of immunoPET diagnostic radioligands to visualize intrabrain Aβ pathology in vivo.

**Methods:**

Three different affinity variants of anti-mouse TfR-binding antibody 8D3, engineered by alanine point mutations, were selected. Bispecific antibodies were designed with knob-into-hole technology with one arm targeting TfR (8D3) and the other arm targeting human Aβ (bapineuzumab). Antibody affinities were measured in an in vitro cell assay. In vivo pharmacokinetic analyses of radioiodinated bispecific antibodies and bapineuzumab in brain, blood and peripheral organs were performed over 7 days post-injection in WT mice and a model of Aβ pathology (*App*^*NL−G−F*^). The strongest TfR affinity bispecific antibody was also evaluated as a positron emission tomography (PET) radioligand for detecting Aβ pathology in WT and *App*^*NL−G−F*^ mice.

**Results:**

The three bispecific antibodies bound to TfR with affinities of 10 nM, 20 nM and 240 nM. Independent of genotype, stronger TfR-affinity resulted in higher initial brain uptake. The two higher-affinity bispecific antibodies behaved similarly and differentiated between WT and *App*^*NL−G−F*^ mice earlier than the lowest affinity variant. Finally, the 10 nM bispecific antibody was able to clearly differentiate between WT and *App*^*NL−G−F*^ mice when used as a PET radioligand.

**Conclusion:**

This study supports the hypothesis that stronger TfR affinity enhances brain uptake at a tracer dose. With the more effective detection of Aβ pathology, stronger TfR affinity is a crucial design feature for future bispecific immunoPET radioligands for intrabrain targets via TfR-mediated transcytosis.

**Supplementary Information:**

The online version contains supplementary material available at 10.1186/s12987-025-00693-2.

## Introduction

Antibodies and antibody fragments make for unique therapeutic and diagnostic tools due to their innate specificity that can be directed to an extensive range of epitopes. The introduction of antibodies and protein-based drugs has considerably improved cancer treatment during the last decades [1, 2]. A similar development is presently seen for brain disorders with two monoclonal antibodies directed towards amyloid-beta (Aβ) emerging as the first disease modifying treatments for Alzheimer’s disease (AD) [3]. However, as large molecules, antibodies do not cross the blood-brain barrier (BBB) efficiently [4–7]. To overcome this obstacle, the molecular Trojan horse strategy can be applied with bispecific antibodies binding to both a central nervous system (CNS) target and a receptor on the BBB. The BBB receptor’s endogenous function is to transport essential molecules into the brain. This strategy allows the bispecific antibody to be carried across the BBB with great efficiency resulting in a several-fold increase in antibody brain concentrations.

The transferrin receptor (TfR) which plays a role in iron homeostasis for the brain was first targeted in the 1990s and has become a common receptor for this strategy [8, 9]. TfR-mediated transport has proven successful in clinical phase I-III trials for delivering enzyme replacement therapies for the lysosomal storage disorder, Hunter syndrome [10–13]. One of these treatments was approved in Japan in 2021 [12]. A phase I trial for delivering Roche’s bispecific anti-Aβ/TfR antibody, Trontinemab, for AD is also on-going [12, 13]. With the shift in the AD field for therapeutic biologics that primarily target a variety of Aβ aggregates, it would be beneficial to have companion diagnostics that detect these various aggregate forms as well. The current gold-standard positron emission tomography (PET) radioligands for detecting amyloid in the living brain are based on small molecules such as carbon-11 (^11^C)-labelled Pittsburgh Compound B ([^11^C]PiB) or other fluorine-18-labelled radioligands that bind to amyloid. These radioligands primarily bind to the dense amyloid core of Aβ plaques. Further, it should be noted that small molecule radioligands also bind to amyloid structures formed by other proteins besides Aβ, e.g. alpha-synuclein that accumulates in Parkinson’s disease or dementia with Lewy bodies [14–16]. Thus, to overcome the limitations of amyloid PET radioligands, bispecific antibody-based PET radioligands for specific detection of Aβ aggregates with the molecular Trojan horse strategy are also being developed. Antibody-based radioligands have shown in rodent Aβ models to be more sensitive than the current small molecule based amyloid radioligands, such as [^11^C]PiB, at detecting changes in Aβ pathology levels during disease progression or as a result of anti-Aβ treatment [17–21].

Over the last couple of decades, research has focused on how to best harness the TfR for brain delivery of large molecular biologics. Several factors have emerged which can influence transcytosis, including: affinity [22–29], valency [5, 26, 29, 30], pH-sensitive binding [31, 32], and size [28]. Affinity has received extensive attention at therapeutic doses (5–50 mg/kg) which tend to be substantially higher than low (tracer) doses (< 1 mg/kg) used for immunoPET imaging of CNS targets. It is well-accepted in the field that a moderate TfR affinity is most optimal for high brain uptake. Antibodies with too high TfR affinity are more likely to be trapped in the capillaries while antibodies with too low affinity do not interact with TfR sufficiently for transcytosis [22, 24, 25, 27]. However, whether this also applies in low doses is currently unclear. In fact, a limited number of studies have suggested that there is a linear relationship between TfR affinity and brain uptake at tracer doses [21–23, 29]. Thus, opposite to the findings at therapeutic dosing, high affinity may be beneficial at low dosing.

Therefore, this study aimed to further investigate the effect of TfR affinity on brain uptake at a tracer dose in wild-type (WT) mice and the *App*^*NL−G−F*^ mouse model of Aβ pathology. Three affinity variants of the rat-anti-mouse TfR1 (mTfR) antibody 8D3 were engineered with single alanine mutations. These TfR-affinity variants were combined with bapineuzumab (Bapi), an antibody that binds Aβ at the N-terminus, with knob-into-hole (KiH) technology to produce bispecific antibodies [33–35]. The brain delivery and pharmacokinetics of the bispecific antibody affinity variants were studied ex vivo and in vivo by PET.

## Methods

### Recombinant antibody production

#### Design

**Fig. 1:**
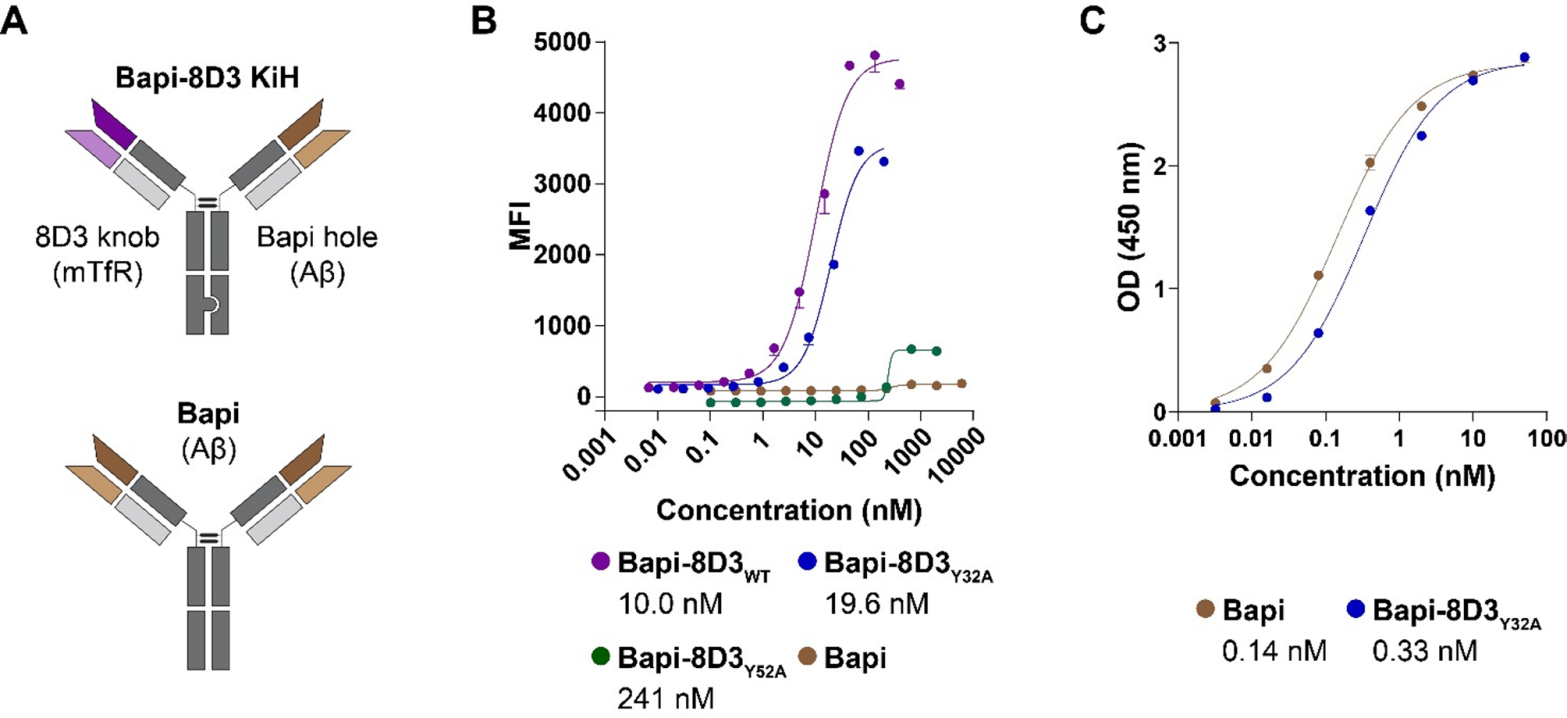
Generation of bispecific antibodies with varying affinity to mTfR. (**A**) Schematic antibodies illustrating the KiH design of bispecific Bapi-8D3 and monospecific Bapi. (**B**) Mean fluorescent intensity (MFI) from the on-cell mTfR affinity assay with Bapi-8D3_WT_, Bapi-8D3_Y32A_, Bapi-8D3_Y52A_, Bapi. EC50 values listed below the legend. (**C**) ELISA analysis of bivalent (Bapi) and monovalent (Bapi-8D3_Y32A_) binding to Aβ with EC50 values listed below the legend

The KiH heavy chain heterodimerization technology was used as a design to generate full-length monovalent, bispecific IgG antibodies based on anti-mTfR, 8D3, and anti-Aβ, Bapi (Fig. 1A). The knob antibody was designed with Fc mutations: S354C/T366W and the hole antibody with Fc mutations: Y349C/T366S/L368A/Y407V [36]. Three affinity variants of 8D3 were designed by introducing single alanine point mutations in the complementarity-determining regions (CDR) of WT 8D3: 8D3_WT_ with no mutations, 8D3_Y32A_ with the Y32A point mutation and 8D3_Y52A_ with the Y52A point mutation [37]. The three affinity-engineered 8D3 knob antibodies were paired with the Bapi-derived hole antibody to generate three Bapi-8D3 bispecific antibodies with varying TfR affinity (Table 1). A monospecific, bivalent Bapi antibody was designed without the KiH technology as a regular IgG. LALA-PG mutations (L234A/L235A/P329G) were introduced to all produced antibodies to reduce effector function [38]. Genes were inserted into pcDNA3.4 plasmids by GenScript Biotech (Leiden, Netherlands) or GeneArt (ThermoFisher Scientific).

**Table 1:** Hole and knob antibody pairings for the KiH antibodies with the 8D3 point mutations and CDR locations

Bispecific antibody	Hole-antibody	Knob-antibody	8D3 point mutation	Point mutation CDR location
Bapi-8D3	Bapi	8D3_WT_	none	n.a.
Bapi-8D3_Y32A_	Bapi	8D3_Y32A_	Y32A	HCDR1
Bapi-8D3_Y52A_	Bapi	8D3_Y52A_	Y52A	HCDR2

CDR: complementarity-determining region; HCDR1: heavy chain complementarity-determining region 1; HCDR3: heavy chain complementarity-determining region 3

#### Production

Bispecific antibodies were generated with separate knob and hole antibody production followed by a disulphide exchange approach (Figure S1). The three 8D3 knob and one Bapi hole antibodies were produced separately with the ExpiCHO Expression System following the manufacturer’s instructions (ThermoFisher Scientific, Stockholm, Sweden). Briefly, ExpiCHO-S cells (ThermoFisher Scientific) were cultured in ExpiCHO Expression medium (ThermoFisher Scientific) at 37 °C, 120 RPM orbital shaking with 80% humidity and 8% CO_2_. ExpiCHO-S cells (6 million viable cells/mL) were transfected with plasmids (1 µg/mL of transfection volume in a 1:1 knob or hole heavy chain plasmid to light chain plasmid ratio) mixed with ExpiFectamine CHO Reagent (ThermoFisher Scientific) and OptiPRO SFM (ThermoFisher Scientific). One day post-transfection, ExpiFectamine CHO Enhancer and ExpiCHO Feed were added to the flask. Cell supernatant was harvested for purification 8 d post-transfection. Monospecific, bivalent Bapi was produced in ExpiCHO-S cells as full antibodies directly.

#### Purification

Knob and hole antibodies and monospecific Bapi were purified from filtered cell supernatant using a HiTrap MabSelect SuRe (Cytiva, Uppsala, Sweden) on an ÄKTA Avant system (Cytiva). A linear gradient of 0.7% acetic acid was used for elution. Following elution, knob and hole antibodies were stored at 4 °C until assembly while the buffer for monospecific Bapi was exchanged to phosphate buffered saline (PBS) with a HiPrep 26/10 Desalting column (Cytiva).

#### Assembly into full-length bispecific antibody

KiH bispecific antibodies were assembled according to Giese and colleagues [35]. Briefly, half antibodies were diluted to 13 µM, adjusted to a pH of 8-8.5 with 120 mM L-arginine, and incubated for 1 h at 35 °C. Following incubation, equal volumes of knob and hole half-antibodies were pooled together and a 200 molar excess of L-reduced glutathione was added. Antibodies incubated for 6 h at 35 °C with end-over-end mixing. The reaction was stopped by exchanging the buffer to PBS with a PD-10 desalting column (Cytiva).

#### Polish

Assembled antibodies were further polished with ion exchange and size exclusion chromatography on an ÄKTA Purifier system (Cytiva). Samples were buffer exchanged to 20 mM TrisHCl, pH 8 with a HiPrep 26/10 desalting column and loaded onto a HiTrap Q HP ion exchange column (Cytiva). The elution buffer was a 20 mM TrisHCl and 1 M NaCl, pH 8 buffer injected from 0 to 50% over 5 column volumes. Collected samples were further purified and buffer exchanged to PBS with a HiLoad 26/600 Superdex 200 pg column (Cytiva).

#### Quality control

Assembled KiH antibody purity was assessed with SDS-PAGE gel with both reducing and non-reducing conditions (Figure S2). Briefly, 1 µg of antibody mixed with Bolt LDS Sample Buffer (ThermoFisher Scientific) with (reducing) or without (non-reducing) NuPAGE Sample Reducing Agent (ThermoFisher Scientific) were heated for 10 min at 90 °C, loaded on a Bolt 4–12% Bis-Tris Protein Gel (ThermoFisher Scientific) and run at 200 V for 35 min with Bolt MES SDS Running Buffer (ThermoFisher Scientific). The gel was fixed in 50% methanol and 7% acetic acid and stained with InstantBlue Coomassie Protein Stain (Abcam, Cambridge, UK).

### On-cell mTfR affinity assay

Affinity to TfR for the bispecific antibody variants was measured with the previously described in vitro cell assay with the exception that Sp2/0-Ag14 cells expressing mTfR endogenously (ATCC, Manassas, USA) were used [39]. With this assay, EC50 values serve as a proxy for apparent affinity because saturation of binding sites on the cell surface is the maximum response being measured. Briefly, Sp2/0-Ag14 cells were cultured in Dulbecco’s Modified Eagle Medium (DMEM, Gibco, Dublin, Ireland) supplemented with 10% Fetal Bovine Serum (FBS, Gibco), 1 mM HyClone Sodium Pyruvate (Cytiva), 2 mM HyClone L-Glutamine (Cytiva) and 1% HyClone Penicillin Streptomycin (Cytiva) in a humidified incubator at 37 °C with 5% CO_2_. The day of the affinity assay, cells were pelleted by centrifuging for 5 min at 1200 RPM, resuspended in PBS and 0.3 million viable cell/well were added to a 96 well U bottom plate. The PBS was discarded and cells were blocked with Fc receptor blocker (NB309, Innovex Biosciences Inc., Richmond, USA) for 20 min. Antibodies were added to the wells in a serial dilution from 400 nM to 6.8 pM for Bapi-8D3_WT_, 600 nM-10.2 pM for Bapi-8D3_Y32A_, and 6000 nM-102 pM for Bapi-8D3_Y52A_ and Bapi. Concentrations were optimized to ensure an upper plateau was achieved for each antibody. Cells were incubated for 30 min at 4 °C, washed with PBS and then incubated with Alexa 488 anti-human IgG1 Fc secondary antibody (1:200 in PBS, H10120, ThermoFisher Scientific) for 30 min at 4 °C. After incubation, cells were washed twice and finally resuspended in 200 µl of PBS. Cells were acquired using a BD FACSLyric Flow Cytometry System (BD Biosciences, San Jose, CA) and data were analysed using flowJo, version 10.8.1 (BD Biosciences). Background corrected mean fluorescent intensity (MFI) was determined by subtracting the MFI of cells incubated with only the secondary antibody from the MFI of cells incubated with both the test antibody and the secondary antibody. The background corrected MFI of two replicates was plotted against the antibody concentration.

### ELISA

Affinity to Aβ for the bivalent and monovalent versions of Bapi were determined using an indirect ELISA, as previously described [21]. Briefly, 96-well half-area plates were coated with Aβ_1−42_ (50 nM in PBS, Innovagen, Lund, Sweden) at 4°C overnight. They were blocked with 1% BSA in PBS for 1 h. Bapi and Bapi-8D3_Y32A_ (50 nM to 3.2 pM) were applied overnight at 4°C. The following day, plates were washed and incubated with horseradish peroxidase (HRP)-conjugated goat anti-human IgG-F(ab’)_2_ (1:2000, #115-035-006, Jackson ImmunoResearch Laboratories, West Grove, USA) for 1 h. Following another wash, the K blue aqueous TMB substrate (Neogen Corp., Lexington, USA) was applied. The reaction was stopped with 1 M H_2_SO_4_. The resulting signal was detected with a spectrophotometer at 450 nm.

### Radiochemistry

The direct iodination Chloramine-T method was used to radiolabel antibodies with iodine-125 (^125^I) (Table 2) [40]. Briefly, 65.3 ± 32.8 µg of antibody was labelled with 10.3 ± 5.9 MBq of [^125^I]NaI stock solution (PerkinElmer Inc., Waltham, USA), by adding 5 µg of Chloramine-T (Sigma Aldrich) in a final reaction volume of 110 µl. The reaction incubated for 90 s at room temperature. Sodium metabisulfite (10 µg, Sigma Aldrich) was added to stop the reaction, bringing the final volume to 120 µL. For iodine-124 (^124^I) labelling, 115 µl [^124^I]NaI stock solution (Advanced Center Oncology Macerata, Montecosaro, Italy) was pre-incubated for 15 min with NaI at a final concentration of 10 µM, then neutralized with 0.5% acetic acid and PBS. After adding 320 µg of antibody, the reaction was initiated by the addition of 40 µg Chloramine-T (Sigma Aldrich) and then quenched with 80 µg sodium metabisulfite (Sigma Aldrich) after 120 s. Radiolabelled antibodies were diluted to 500 µL with PBS, purified using a NAP-5 size exclusion column (Cytiva) to remove any free iodine and eluted in PBS. Indirect ELISAs were performed as quality control to ensure that the radiolabelled antibodies had retained their binding affinity to mTfR (1 µg/ml for Bapi-8D3_WT_ and Bapi-8D3_Y32A_; 10 µg/ml for Bapi-8D3_Y52A_) and Aβ (50 nM) (Figure S3).

**Table 2:** Radiochemical reaction yield and specific activity of radioligands

Radioligand	Radiochemical yield (%)	Molar activity (MBq/nmol)
[^124^I]I-Bapi-8D3_WT_	83.4	16.1
[^125^I]I-Bapi-8D3_WT_	78.0 ± 6.4	18.4 ± 3.6
[^125^I]I-Bapi-8D3_Y32A_	88.7 ± 1.6	23.3 ± 1.1
[^125^I]I-Bapi-8D3_Y52A_	81.2 ± 7.7	19.6 ± 6.6
[^125^I]IBapi	85.7 ± 5.2	23.8 ± 1.9

### Mice

Ex vivo biodistribution studies and PET imaging were performed on 12–18 month old WT C57BL/6 mice (*n* = 53) and *App*^*NL−G−F*^ mice (*n* = 53). The *App*^*NL−G−F*^ model is a knock-in model which expresses mouse amyloid precursor protein (APP) with a humanized Aβ sequence harbouring the Swedish (*KM670/671NL*), Arctic (*E693G*), and Iberian (*I716F*) mutations. This model displays aggressive amyloidosis with Aβ plaque pathology beginning around 3 months and consisting mainly of Aβ_1−42_ [41, 42]. Both males and females were included. The number of mice for each experiment and the dose of each antibody are given in Table S1. All procedures involving mice were approved by the Uppsala County Animal Ethics board (5.8.18–20401/2020) and carried out in accordance with the Swedish Animal Welfare Agency regulations and the European Communities Council Directive of 22 September 2010 (2010/63/EU). Mice were housed in an approved animal facility at Uppsala University with free access to food and water.

### Pharmacokinetics and ex vivo biodistribution

WT or *App*^*NL−G−F*^ mice under mild isoflurane sedation (Baxter Medical AB, Kista, Sweden) received a tracer dose (0.21 ± 0.02 mg/kg, 1.00 ± 0.20 MBq) of [^125^I]I-Bapi-8D3_WT_, [^125^I]I-Bapi-8D3_Y32A_, [^125^I]I-Bapi-8D3_Y52A_, or [^125^I]I-Bapi injected intravenously. Blood (8 µL) was sampled from the tail vein 1, 2, 4, 6, 24, 48, 72 and 168 h post-injection.

Prior to euthanasia, mice were anaesthetized with isoflurane and a terminal blood sample was collected from the heart. Half of the terminal blood sample was separated into plasma and blood cell pellet by centrifugation at 10,000 x g for 5 min. Mice were euthanized at 4, 24, 72 and 168 h post-injection via transcardial perfusion with 40 mL of 0.9% NaCl for 2.5 min. Immediately after, the left cortex was collected for capillary depletion and the right hemisphere, left midbrain and left cerebellum were frozen on dry ice. Lung, heart, liver, pancreas, spleen, kidney, femoral muscle, femoral bone, skull and thyroid were harvested and a urine sample was collected. Radioactivity in all samples was measured by γ-counting (2480 Wizard™, PerkinElmer). Antibody concentrations were expressed as percent of the injected dose of activity per gram tissue:

%ID/g = measured radioactivity per gram tissue / total injected dose of radioactivity x 100.

### Capillary depletion

Perfused left cortices underwent capillary depletion immediately after perfusion as previously described [28]. Briefly, cortices were homogenized with 6 strokes in an ice-cold Dounce homogeniser in 0.4 mL cold physiological buffer (10 mM HEPES, 141 mM NaCl, 4 mM KCl, 2.8 mM CaCl_2_, 1 mM MgSO_4_, 1 mM NaH_2_PO_4_, 10 mM D-glucose, pH 7.4) before adding 0.8 ml of 30% Ficoll 400 (Sigma Aldrich) and homogenizing with 2 more strokes. Homogenates were centrifuged at 3900 g for 30 min at 4 °C. The two fractions, a capillary enriched pellet and a parenchymal supernatant, were separated, and the radioactivity in each fraction was measured by γ-counting (PerkinElmer).

### Ex vivo autoradiography

One mouse from each time point was randomly selected and the right hemisphere from these mice were cryosectioned (CM1850, Leica Biosystems, Nussloch, Germany) into 20 μm sagittal sections. Two sections per mouse were exposed to a phosphor imaging plate (MS, MultiSensitive, PerkinElmer) for 10 days. Plates were scanned in a Typhoon phosphor imager (Cytiva). The resulting digital images were decay corrected to the day of injection, normalized to the injected dose and converted to a false colour scale (Royal) in ImageJ.

### Aβ immunohistochemistry

Immunohistochemistry was performed on sections from mice euthanized at 72 h post-injection. Brain sections were fixed in 4% paraformaldehyde and washed in PBS. Sections were boiled in 25 mM citrate buffer (pH 7.3) for 10 s and left to cool to room temperature for 20 min before being treated with 70% formic acid (FA) for 5 min. Following the formic acid treatment, sections were rinsed in milliQ water and washed in PBS. Endogenous peroxidase was blocked with 20 min exposure to 3% hydrogen peroxide. Since a mouse primary antibody was used for staining mouse tissue, the M.O.M. Mouse Ig Blocking Reagent (Vector Laboratories Inc., California, USA) was used according to the manufacturer’s instructions. Sections were permeabilized with 0.4% triton X-100 in PBS for 5 min. The primary antibody, 3D6 (murine version of Bapi, produced in-house [43]), was diluted to 10 µg/ml in 0.1% Tween-20 in PBS supplemented with MOM mouse diluent (Vector Laboratories Inc.) and added to the sections overnight at 4 °C. The next day, sections were washed in PBS and the secondary goat-anti-mouse-biotin antibody (1:250 in PBS, BA-9200-15, Vector Laboratories Inc.) was added for 45 min at room temperature. Sections were washed before incubating with streptavidin-HRP (1:500 in PBS; Mabtech AB, Nacka Strand, Sweden) for 45 min. After another wash, the peroxidase substrate from the NovaRED Substrate Kit (Vector Laboratories Inc.) was applied for 3 min following the manufacturer’s instructions. Sections were washed again, counterstained with haematoxylin and rinsed in tap water for 5 min. Then sections were dehydrated in increasing concentrations of ethanol and xylene before mounting with Pertex mounting medium (Histolab, Stockholm, Sweden). Brightfield images were acquired with a Zeiss Observer Z1 microscope (Carl Zeiss Imaging GmBH, Jena, Germany) and processed using ZEN 3.7 software (Carl Zeiss Imaging GmBH).

### PET imaging

For PET imaging, WT or *App*^*NL−G−F*^ mice were intravenously administered with [^124^I]I-Bapi-8D3_WT_ (1.15 ± 0.01 mg/kg, 4.19 ± 0.50 MBq) under mild isoflurane sedation (Baxter). At 72 h post-administration, mice underwent a 90 min PET scan (Mediso NanoPET/MR, Mediso Medical Imaging System, Hungary) followed by a 5 min CT (Mediso NanoSPECT/CT, Mediso Medical Imaging System). Anaesthesia was maintained during scanning with 3.5–4.0% sevoflurane in a 0.5 L/min flow of 50% oxygen and 50% medical air. After scanning, the mice were perfused, the brain was isolated and the radioactivity in the brain was measured by γ-counting (PerkinElmer) according to the same procedure as described above for ex vivo studies. The PET data was reconstructed on a 160 × 160 × 128 grid with 0.5 × 0.5 × 0.6 mm^3^ voxels using 3-dimensional ordered-subsets expectation maximisation (20 iterations). The CT raw files were reconstructed using filtered back-projection. Processing of the PET and CT images was performed with Amide, version 1.0.4 [44]. The CT scans were manually aligned with a T2-weighted, MRI-based mouse brain atlas containing outlined regions of interest (ROIs) [45]. The PET images were subsequently aligned with the CT image containing the brain atlas ROIs. The average activity in each ROI was converted to %ID/g brain, assuming that one cm^3^ corresponded to one gram of brain tissue.

### Statistical analyses

GraphPad Prism 9.4.1 was used for statistical analyses. The EC50 values for the on-cell affinity assay and ELISA were calculated from agonist concentration vs. response curves with variable slope (four parameters). Results are reported as mean ± standard deviation, except for the area under the concentration curve (AUC) graphs which are the mean ± standard error. The whole blood half-life was calculated using a two-phase decay model and Y0 was set to 50% as previously described [28]. The half-life for each antibody and mouse genotype was expressed as the mean ± standard deviation based on mice that were euthanized at 3 and 7 days post-injection. A two-way ANOVA with Šídák’s multiple comparisons test was performed to determine at which time point the brain concentration of each antibody significantly differed between WT and *App*^*NL−G−F*^ mice in ex vivo studies, and to assess regional concentration differences between WT and *App*^*NL−G−F*^ mice in the PET studies. An unpaired t-test was used to compare ex vivo measured whole brain concentrations between WT and *App*^*NL−G−F*^ in PET-scanned mice. Differences between brain and blood pharmacokinetic AUCs were assessed with Brown-Forsythe and the Welch ANOVA tests with Dunnett’s T3 multiple comparisons test. All other data were not subjected to statistical testing, and any reported differences should be interpreted as observed trends rather than statistically confirmed effects.

## Results

### Alanine point mutations yielded three mTfR affinity variants

Bispecific Bapi-8D3 antibodies and Bapi were produced with the KiH design (Fig. 1A, S2). In an on-cell mTfR affinity assay, the EC50 of Bapi-8D3_Y32A_ and Bapi-8D3_Y52A_ was 2-fold and 24-fold higher, respectively, than the EC50 of Bapi-8D3_WT_ (Fig. 1B, Table S2). Bapi also bound to Aβ with a sub-nanomolar EC50 in both the bivalent (Bapi) and monovalent (Bapi-8D3_Y32A_) formats (Fig. 1C).

### Bispecific antibodies with stronger TfR-affinity had higher brain uptake at a tracer dose

**Fig. 2:**
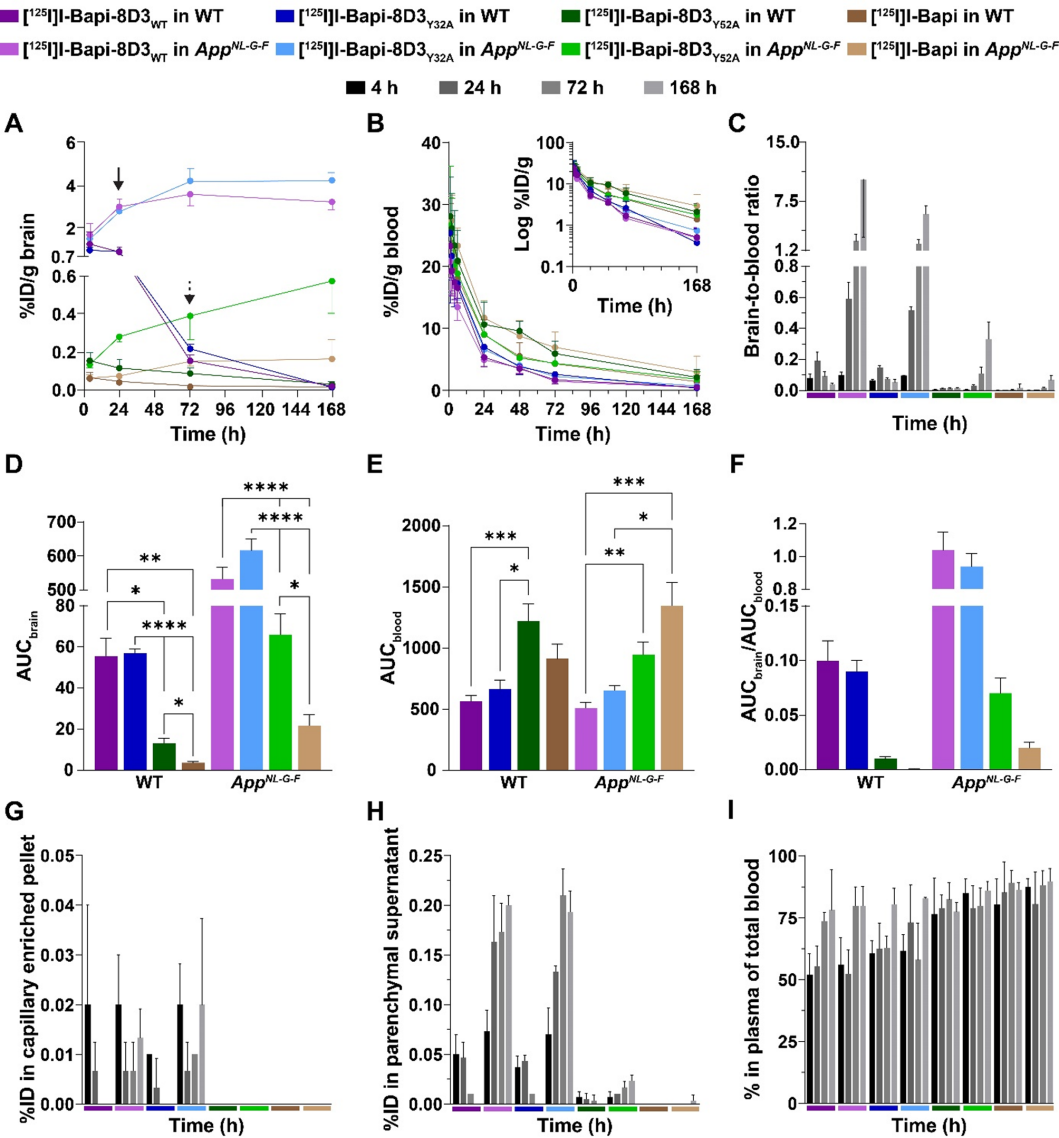
Ex vivo biodistribution of three bispecific affinity variants and Bapi in WT and *App*^*NL−G−F*^ mice over 168 h post-injection. (**A**) Brain concentrations (%ID/g) of [^125^I]I-Bapi-8D3_WT_, [^125^I]I-Bapi-8D3_Y32A_, [^125^I]I-Bapi-8D3_Y52A_ and [^125^I]I-Bapi in WT and *App*^*NL−G−F*^ mice 4, 24, 72, and 168 h post-injection. The first time when the concentration in *App*^*NL−G−F*^ mice is significantly higher than the concentration in WT mice is indicated by a solid arrow for [^125^I]I-Bapi-8D3_WT_ and [^125^I]I-Bapi-8D3_Y32A_ and a dashed arrow for [^125^I]I-Bapi-8D3_Y52A_ and [^125^I]I-Bapi. (**B**) Whole blood pharmacokinetics (%ID/g of blood) over 168 h post-injection. (**C**) Brain-to-blood ratio in WT and *App*^*NL−G−F*^ mice 4, 24, 72, and 168 h post-administration. AUC from the brain (AUC_brain_, **D**) and blood (AUC_blood_, **E**) pharmacokinetic curves of each antibody in WT and *App*^*NL−G−F*^ mice (* *p* ≤ 0.05, ** *p* ≤ 0.01, *** *p* ≤ 0.001, **** *p* < 0.0001). (**F**) The ratio of AUC_brain_/AUC_blood_ for each antibody in WT and *App*^*NL−G−F*^ mice. Concentrations (%ID) in the capillary enriched pellet (**G**) and parenchymal supernatant (**H**) from capillary depletion of cortices at 4, 24, 72 and 168 h post-administration. (**I**) Percent in plasma fraction of total blood in WT and *App*^*NL−G−F*^ mice 4, 24, 72, and 168 h post-administration

To assess how antibodies with a TfR-binding modality with different affinities behave in vivo, each antibody was radiolabelled with ^125^I and injected into mice at a tracer dose of 0.2 mg/kg. The brain concentrations of the stronger TfR binders, [^125^I]I-Bapi-8D3_WT_ and [^125^I]I-Bapi-8D3_Y32A_, were similar 4 h post-injection (Fig. 2A), 6- to 13-fold higher than [^125^I]I-Bapi-8D3_Y52A_, and 16- to 27-fold higher than [^125^I]I-Bapi. The concentration in the brain 4 h post-injection was similar between WT and *App*^*NL−G−F*^ mice for each antibody. Over time, the concentration of injected antibody in the brain decreased in WT mice and increased in *App*^*NL−G−F*^ mice. These trends occurred in both the cerebrum and cerebellum (Figure S4). The first time point in which the brain concentration of antibody was significantly different between WT and *App*^*NL−G−F*^ mice was 24 h post-injection for [^125^I]I-Bapi-8D3_WT_ (*p* < 0.001) and [^125^I]I-Bapi-8D3_Y32A_ (*p* < 0.001) and 72 h for [^125^I]I-Bapi-8D3_Y52A_ (*p* < 0.01) and [^125^I]I-Bapi (*p* < 0.01, Fig. 2A). The blood kinetics were comparable between WT and *App*^*NL−G−F*^ mice for each antibody and showed a trend that stronger affinity to mTfR resulted in quicker blood elimination (Fig. 2B; Table 3).

The brain-to-blood ratio for each antibody was similar between *App*^*NL−G−F*^ and WT mice 4 h post-injection (Fig. 2C). In WT mice, the brain-to-blood ratio peaked at 24 h for [^125^I]I-Bapi-8D3_WT_ and [^125^I]I-Bapi-8D3_Y32A_, while it stayed constantly low for [^125^I]I-Bapi-8D3_Y52A_ and [^125^I]I-Bapi over the 168 h post-injection study period. The brain-to-blood ratio in *App*^*NL−G−F*^ mice continued to increase until the final time point for each antibody. At 168 h post-injection, the brain-to-blood ratio for the bispecific antibody with the highest TfR affinity, [^125^I]I-Bapi-8D3_WT_, was 2-fold higher, 31-fold higher and 150-fold higher than the ratios observed for [^125^I]I-Bapi-8D3_Y32A_, [^125^I]I-Bapi-8D3_Y52A_, and [^125^I]I-Bapi, respectively.

**Table 3:** Elimination half-life (h) in whole blood

	WT	App^NL−G−F^
[^125^I]I-Bapi-8D3_WT_	18.0 ± 5.5	16.7 ± 5.3
[^125^I]I-Bapi-8D3_Y32A_	15.2 ± 2.2	15.3 ± 2.9
[^125^I]I-Bapi-8D3_Y52A_	34.4 ± 19.8	21.7 ± 10.6
[^125^I]I-Bapi	26.0 ± 9.7	22.5 ± 8.8

Data presented as mean ± standard deviation

The ranking of AUC_brain_ for the antibodies was the same in both WT and *App*^*NL−G−F*^ mice (Fig. 2D). The AUC_brain_ for [^125^I]I-Bapi-8D3_WT_ and for [^125^I]I-Bapi-8D3_Y32A_ did not significantly differ from each other (WT: *p* > 0.999; *App*^*NL−G−F*^: *p* = 0.654). The AUC_brain_ for both [^125^I]I-Bapi-8D3_WT_ and [^125^I]I-Bapi-8D3_Y32A_ were significantly higher than the AUC_brain_ for [^125^I]I-Bapi-8D3_Y52A_ (WT: 4-fold, *p* < 0.05; *App*^*NL−G−F*^: 8 to 9-fold, *p* < 0.001). The AUC_brain_ for [^125^I]I-Bapi-8D3_Y52A_ was significantly higher than the AUC_brain_ for [^125^I]I-Bapi (WT: 4-fold, *p* < 0.05; *App*^*NL−G−F*^: 4-fold, *p* < 0.05).

Overall, the AUC_blood_ for each antibody did not differ between WT or *App*^*NL−G−F*^ mice (Fig. 2E). The statistical analysis of the AUC_blood_ supported the trend that stronger affinity to mTfR resulted in quicker blood elimination.

The ratio of AUC_brain_ and AUC_blood_ (AUC_brain_/AUC_blood_) was always higher in *App*^*NL−G−F*^ mice than in WT mice (11-fold for both [^125^I]I-Bapi-8D3_WT_ and [^125^I]I-Bapi-8D3_Y32A_; 7-fold for [^125^I]I-Bapi-8D3_Y52A_; 4-fold for [^125^I]I-Bapi; Fig. 2F). Independent of genotype, the AUC_brain_/AUC_blood_ ratio for [^125^I]I-Bapi-8D3_WT_ was similar to the ratio for [^125^I]I-Bapi-8D3_Y32A_. Both of these ratios were 8- to 15-fold higher than the ratio for [^125^I]I-Bapi-8D3_Y52A_ and 21- to 65-fold higher than [^125^I]I-Bapi.

Capillary depletion was performed to further investigate the fraction of radiolabelled antibodies that reached the brain parenchyma and the fraction that was retained in the capillaries, likely as a consequence of binding to TfR expressed by the endothelial cells of the BBB (Fig. 2G, H). In the capillary enriched pellet, there were low levels of [^125^I]I-Bapi-8D3_WT_ and [^125^I]I-Bapi-8D3_Y32A_ at every time point and undetectable levels of low TfR-affinity [^125^I]I-Bapi-8D3_Y52A_ and non-TfR-binding [^125^I]I-Bapi (Fig. 2G). The antibody concentrations in the parenchymal fraction reflected the total brain pharmacokinetics well (Fig. 2H). Overall, [^125^I]I-Bapi-8D3_WT_ and [^125^I]I-Bapi-8D3_Y32A_ parenchymal concentrations were higher than the concentration of [^125^I]I-Bapi-8D3_Y52A_ while the parenchymal concentration of [^125^I]I-Bapi was below the detection limits. At 4 h post-injection, there were similar parenchymal levels of each antibody in WT and *App*^*NL−G−F*^ mice. Over the 168 h post-injection, the parenchymal concentrations of each antibody then decreased in WT mice and increased in *App*^*NL−G−F*^ mice. Even the parenchymal concentration of [^125^I]I-Bapi was detectable in *App*^*NL−G−F*^ mice at 168 h post-injection.

At 4 h post-injection in both WT and *App*^*NL−G−F*^ mice, the percentage of radioactivity in plasma (relative to total blood) for the stronger TfR binders, [^125^I]I-Bapi-8D3_WT_ and [^125^I]I-Bapi-8D3_Y32A_, tended to be lower than for [^125^I]I-Bapi-8D3_Y52A_ and [^125^I]I-Bapi (Fig. 2I). The percentage of [^125^I]I-Bapi-8D3_WT_ and [^125^I]I-Bapi-8D3_Y32A_ in plasma increased over time so that at 168 h post-injection, the percentage in plasma was similar for all antibodies in both genotypes.

Peripherally, the antibodies behaved similarly in WT and *App*^*NL−G−F*^ mice (Figure S5). All three radiolabelled bispecific TfR-binding antibodies had higher concentrations in the spleen than [^125^I]I-Bapi, the control without a TfR-binding domain, at 4 h post-injection. The level in the spleen also reflected the bispecific antibody affinity to TfR such that the concentration in the spleen of [^125^I]I-Bapi-8D3_WT_ was higher than [^125^I]I-Bapi-8D3_Y32A_ which was higher than [^125^I]I-Bapi-8D3_Y52A_. The concentrations of [^125^I]I-Bapi-8D3_WT_ and [^125^I]I-Bapi-8D3_Y32A_ were also higher than the concentrations of [^125^I]I-Bapi-8D3_Y52A_ and [^125^I]I-Bapi in both the bone and skull at 4 h post-injection. Over time, the concentration in peripheral organs decreased for all antibodies in both WT and *App*^*NL−G−F*^ mice.

**Fig. 3:**
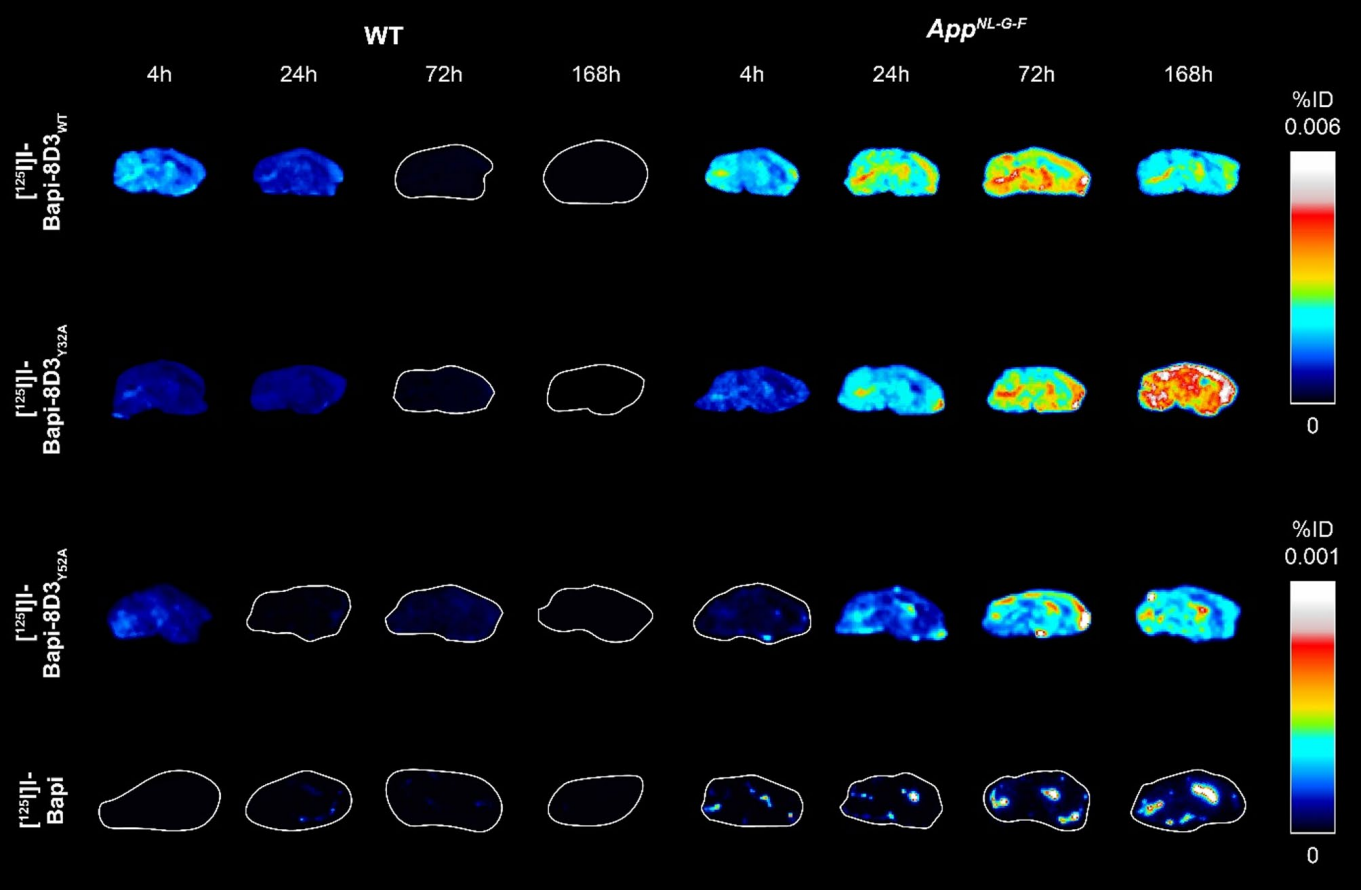
Ex vivo autoradiography of three bispecific affinity variants and Bapi in WT and *App*^*NL−G−F*^ mice over 168 h post-injection. Autoradiography in sagittal sections from WT and *App*^*NL−G−F*^ mice injected with [^125^I]I-Bapi-8D3_WT_, [^125^I]I-Bapi-8D3_Y32A_, [^125^I]I-Bapi-8D3_Y52A_ and [^125^I]I-Bapi at 4, 24, 72 and 168 h post-injection. The intensity for [^125^I]I-Bapi-8D3_WT_ and [^125^I]I-Bapi-8D3_Y32A_ are scaled differently than [^125^I]I-Bapi-8D3_Y52A_ and [^125^I]I-Bapi

### Stronger TfR-affinity binders had a more even distribution in the *App*^*NL-G-F*^ brain that matched Aβ pathology

Autoradiography performed on sections from mice injected with each antibody and sacrificed at the different time points illustrated the trends from the ex vivo biodistribution (Fig. 3). The radioactive signal increased in *App*^*NL−G−F*^ sections and decreased in WT sections over the 168 h post-injection. There was higher overall signal for the stronger TfR binders, [^125^I]I-Bapi-8D3_WT_ and [^125^I]I-Bapi-8D3_Y32A_, than for [^125^I]I-Bapi-8D3_Y52A_ and [^125^I]I-Bapi.

**Fig. 4:**
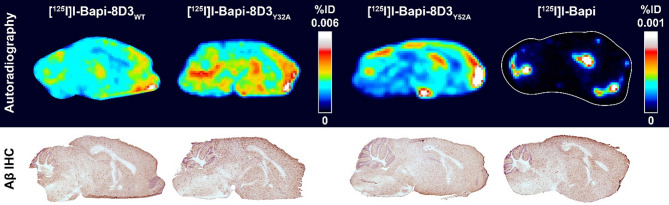
More even antibody distribution with higher affinity to mTfR. Ex vivo autoradiography and Aβ immunohistochemistry (IHC) in sections from *App*^*NL−G−F*^ mice 72 h post-injection of [^125^I]I-Bapi-8D3_WT_, [^125^I]I-Bapi-8D3_Y32A_, [^125^I]I-Bapi-8D3_Y52A_ and [^125^I]I-Bapi. The intensity for [^125^I]I-Bapi-8D3_WT_ and [^125^I]I-Bapi-8D3_Y32A_ are scaled differently than [^125^I]I-Bapi-8D3_Y52A_ and [^125^I]I-Bapi

The autoradiography further illustrated the distribution of radiolabelled antibody within the brain. [^125^I]I-Bapi-8D3_WT_ and [^125^I]I-Bapi-8D3_Y32A_ distributed evenly throughout the brain while [^125^I]I-Bapi localized in hotspots. The distribution of [^125^I]I-Bapi-8D3_Y52A_ was a mix of both distribution types. It had a uniform (although low) signal throughout the brain and the hotspots near the ventricles (Figs. 3 and 4).

Immunohistochemical staining of Aβ confirmed that there was extensive pathology throughout the brains of *App*^*NL−G−F*^ mice at this age, including in the hippocampus, cortex, and cerebellum (Fig. 4). The autoradiography signal from the bispecific antibodies 72 h post-injection co-localized well with pathology throughout the entire brain section. One radioactive hotspot in the sections from mice injected with [^125^I]I-Bapi-8D3_Y52A_ or [^125^I]I-Bapi co-localized with the pathology in the hippocampus but overall, the radioactive hotspots did not properly reflect Aβ pathology throughout the brain section.

### PET-imaging differentiates between *App*^*NL-G-F*^ and WT mice

**Fig. 5:**
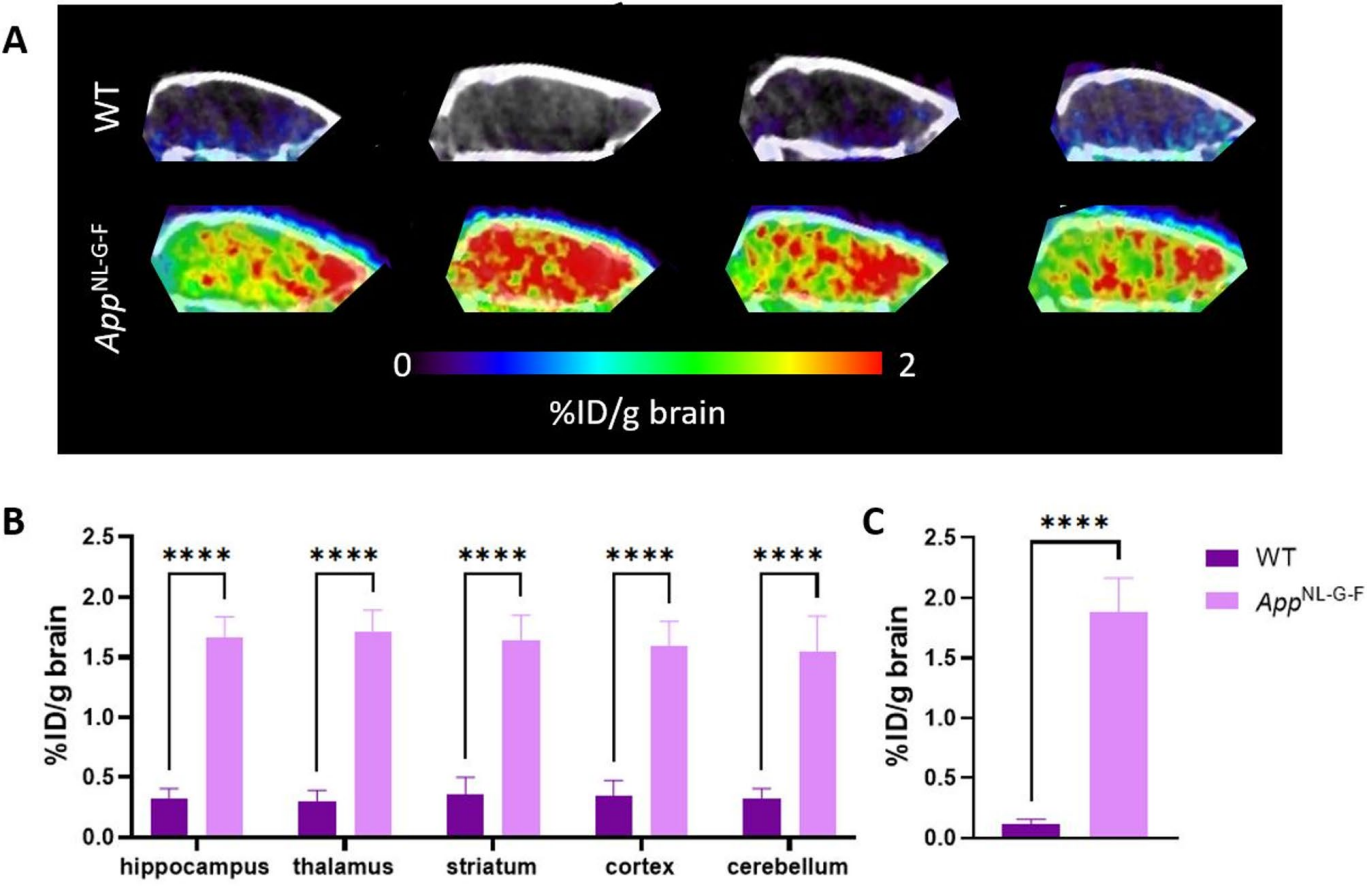
PET imaging reveals more intense signal in *App*^*NL−G−F*^ _mice compared to WT. (**A**) Sagittal PET/CT images acquired at 72 h post administration of [^124^I]I-Bapi-8D3_WT_. The antibody was retained more in the brain of the *App*^*NL−G−F*^ mice with Aβ pathology than in WT mice without pathology demonstrating that it could be used as a diagnostic tool to visualize the regional distribution of Aβ pathology in the living brain. (**B**) Quantification of [^124^I]I-Bapi-8D3_WT_ brain concentrations based on PET images in different brain regions expressed as %ID/g brain. (**C**) Brain concentrations of [^124^I]I-Bapi-8D3_WT_ based on gamma counting and expressed as %ID/g brain. (* *p* ≤ 0.05, ** *p* ≤ 0.01, *** *p* ≤ 0.001, **** *p* < 0.0001)

PET images acquired 72 h post-administration of [^124^I]I-Bapi-8D3_WT_ showed a significantly higher signal in *App*^NL−G−F^ mice compared to WT mice (Fig. 5A). Consistent with previous ex vivo gamma counting results obtained with the ^125^I-labelled version, image-based quantification indicated [^124^I]I-Bapi-8D3_WT_ brain concentrations of around 2%ID/g brain in most brain regions of *App*^NL−G−F^ mice (Fig. 5B). This was further confirmed by ex vivo gamma counting of isolated brains post-PET (Fig. 5C). In WT mice, brain concentrations of [^124^I]I-Bapi-8D3_WT_ were 0.3%ID/g brain based on PET quantification in the living brain, and 0.1%ID/g brain based on ex vivo gamma counting of perfused brains after PET scanning.

## Discussion

Three Bapi-8D3 bispecific antibodies with different affinities to mTfR were used to study how TfR affinity influences brain uptake in WT and *App*^*NL−G−F*^ mice at a tracer dose. The effects of affinity on transcytosis have been thoroughly studied at therapeutic doses but few studies have explored the effects at lower doses [21–24, 27]. Understanding TfR-mediated transcytosis at tracer doses is becoming more relevant with the development of bispecific antibody-based PET radioligands for diagnosing CNS diseases [15, 17, 46–49]. At a tracer dose of 0.2 mg/kg, higher affinity to TfR resulted in higher brain uptake than lower TfR affinity. Despite the 2-fold difference in affinity in vitro, the two stronger TfR-binding bispecific antibodies, [^125^I]I-Bapi-8D3_WT_ and [^125^I]I-Bapi-8D3_Y32A_, had similar in vivo brain pharmacokinetics. Their initial brain uptake (%ID/g brain) and overall brain exposure (AUC_brain_) were higher than the weaker bispecific antibody, [^125^I]I-Bapi-8D3_Y52A_; which in turn had higher initial brain uptake and overall brain exposure than the control [^125^I]I-Bapi without TfR affinity. The capillary depletion further confirmed that successful transcytosis of antibodies into the brain had occurred and that the differences in brain uptake were primarily driven by changes in parenchymal antibody concentrations. This trend supports the proposed hypothesis in literature that the effects of TfR affinity on brain transcytosis are dose-dependent, i.e. that higher affinity results in increased brain delivery at low doses [21–23, 29].

The similar pharmacokinetic profiles between [^125^I]I-Bapi-8D3_WT_ and [^125^I]I-Bapi-8D3_Y32A_ were also consistent with previous findings in literature. Yu and colleagues tested four antibodies with varying affinities against TfR: 1.7 nM, 6.9 nM, 65 nM and 111 nM [22]. The antibody with 1.7 nM affinity had the highest brain concentrations 4 h post-injection and the 111 nM affinity antibody had the lowest. In the middle, the 6.9 and 65 nM affinity antibodies followed the same brain concentration profiles over 24 h post-injection. The affinities of the two stronger TfR-binding bispecific antibodies developed in the present study were also within this affinity range. Therefore, while there appears to be a linear relationship between affinity and transcytosis efficiency at tracer doses, there seems to be an affinity range between approximately 5 and 65 nM where this effect plateaus. One limitation of this study was the affinity range of the three 8D3 variants. Since the strongest affinity variant was the WT form of 8D3, it was not possible to test affinities stronger than 10 nM with the monovalent design. An 8D3 variant with nanomolar or sub-nanomolar affinity and a variant with an affinity around 100 nM (i.e. between Bapi-8D3_Y32A_ and Bapi-8D3_Y52A_) would be beneficial to further investigate the effects of affinity on TfR-binding bispecific antibody pharmacokinetics at a tracer dose.

The binding strength to TfR impacted the distribution of the four antibodies in blood and to peripheral organs in both WT and *App*^*NL−G−F*^ mice such that there was quicker elimination from blood and higher peripheral uptake with stronger binding. In fact, the weakest TfR-binding bispecific antibody, [^125^I]I-Bapi-8D3_Y52A_, had a similar blood exposure (AUC_blood_) to [^125^I]I-Bapi, the control antibody that had no binding to TfR. This relationship aligned well with previous studies [6, 21, 23, 50, 51]. Peripheral tissue with high TfR expression levels such as the spleen, bone and skull also showed a direct relationship between affinity and tissue concentrations, especially 4 h post-injection. Clearance from blood is mediated by binding to TfR at the BBB and on peripheral organs and thus, a stronger affinity to TfR increases the availability of the antibody to interact with tissue outside the blood.

Size and valency have also been suggested to influence transcytosis efficiency [28–30, 52]. Therefore, the KiH design used here offers two advantages. Firstly, it ensured uniform size amongst all four antibodies. Another advantage is that this design allowed for monovalent binding to TfR. Monovalent binding is considered optimal when hijacking the TfR for BBB transportation because multivalent binding has been shown to cause TfR clustering and lysosomal degradation of both TfR and the antibody [26, 30].

Stronger TfR affinity also led to more sensitive detection of Aβ pathology. For each antibody, the total brain and parenchymal concentrations 4 h post-injection were the same between WT and *App*^*NL−G−F*^ mice. The concentrations in both total brain and parenchyma proceeded to increase in *App*^*NL−G−F*^ mice and decrease in WT mice over the following 164 h. The discrepancy in pharmacokinetic profiles between genotypes is attributable to the presence of extracellular Aβ deposits in *App*^*NL−G−F*^ mice. Upon entering the brain parenchyma, antibodies targeting the TfR also bind to neuronal TfR, leading to internalization and subsequent intracellular catabolism. This process is more pronounced in WT mice, which lack Aβ deposits, allowing for greater antibody uptake and degradation. Because iodine-125 is a non-residualizing radionuclide, it is released from the cells following catabolism, resulting in a gradual decline in the radioactive signal over time, as observed in the WT mice in this study. This phenomenon has been clearly demonstrated in previous dual labelling studies [23, 46, 53, 54]. TfR-binding antibodies labelled with a residualizing radionuclide, such as radiometals (e.g., copper-64, zirconium-89, or indium-111), and a non-residualizing radionuclide, such as iodine-125, have shown that residualizing radionuclides accumulate in neurons over time, while non-residualizing radionuclides are gradually excreted from the tissue. However, a bispecific antibody with higher affinity for its intraparenchymal target than for TfR will preferentially bind to the parenchymal target upon crossing the BBB. This targeted accumulation limits internalization and degradation within neurons. Similar brain concentration profiles have been observed with bispecific antibodies targeting TfR and other intraparenchymal proteins, such as myelin oligodendrocyte glycoprotein [55].

Despite similarly shaped brain concentration profiles, the two bispecific antibodies with stronger TfR affinity could differentiate between genotypes earlier than the weakest bispecific antibody and control antibody. Stronger TfR affinity also resulted in better target engagement with Aβ pathology as seen with the autoradiography and immunohistochemical stainings. The control antibody showed hotspots characteristic of antibodies without a TfR-binding modality [6, 7, 43, 50]. The stronger the affinity to TfR, the more evenly distributed the autoradiography signal was and the better the autoradiography signal reflected the immunohistochemically stained Aβ pathology. Altogether, the ex vivo results suggested that low dosing, combined with high affinity for TfR, promotes rapid and efficient accumulation of the bispecific antibody at the target site. At the same time, this approach contributes to the decline in radioiodine signal in the brains of WT mice, which might otherwise be masked by ongoing brain uptake at higher doses or with lower-affinity constructs. This strategy therefore enhances the contrast between the presence and absence of an extracellular antigen such as parenchymal Aβ. Moreover, it is likely to increase the sensitivity of antibody-based PET radioligands for detecting early Aβ pathology, and strongly supports the use of this approach in molecular imaging applications, particularly when the CNS target is extracellular.

Consistent with this reasoning, in vivo imaging using the highest-affinity TfR-binding bispecific antibody as an immunoPET radioligand achieved unparalleled visualization of Aβ pathology in the brains of *App*^NL−G−F^ mice. In contrast, WT mice lacking Aβ showed low radioligand retention. Notably, in vivo PET quantification of radioligand concentrations in the brains of WT mice indicated higher levels compared to those measured ex vivo by gamma counting of perfused brains. This discrepancy arises because the living brain contains approximately 5% blood, and the radioligand in the brain’s blood volume enhances the PET signal in WT mice. This is a crucial consideration when selecting compounds for immunoPET applications as rapid clearance from the blood is likely to improve the distinction between brains with Aβ pathology and those without. This is another important difference from therapeutic constructs, where longer circulation times are typically preferable. Furthermore, the use of different radionuclides may lead to different imaging results [54] and thus, brain delivery of an immunoPET radioligand with different radiolabels may benefit from optimizing the brain uptake kinetics via TfR affinity optimization.

Previous studies of bispecific antibodies with maintained effector functions have displayed the ability to differentiate between WT mice and mice that express an intrabrain target such as Aβ. Despite this, these bispecific antibodies have consistently shown decreasing target bound concentrations in the brain over time after a tracer dose injection [5, 17, 21, 22, 50]. In the present study, antibody concentrations continued to increase in the brains of mice with Aβ brain pathology during the full 168 h post-injection study period. The loss of effector function that was achieved by the insertion of the LALA-PG mutations and the efficient transcytosis due to monovalent TfR-binding likely contributed to the observed absence of net-elimination. In line with this, the brain concentrations of bispecific antibodies with monovalent TfR-binding and reduced effector function (LALA/G237A or LALA/K322A mutations) or no Fc at all (a di-scFv design) decreased in WT mice while they initially increased or plateaued in transgenic Aβ mouse models over time after tracer dose injections [7, 19, 46]. In addition, elimination of effector function is also a beneficial design trait for bispecific antibody-based PET ligands to avoid both central and peripheral immune responses.

In conclusion, the use of KiH-format bispecific anti-Aβ/mTfR antibodies with varying TfR affinities supports the hypothesis that higher TfR affinity leads to increased brain uptake at tracer doses. Furthermore, bispecific antibodies with stronger TfR affinity differentiated between WT and *App*^*NL−G−F*^ mice earlier and visualized Aβ pathology more distinctively than the bispecific antibody with weaker or no TfR affinity. Loss of antibody effector function potentially reduced the elimination from brain. ImmunoPET imaging using a low dose of the bispecific antibody with the highest TfR affinity showed intense signals in *App*^*NL−G−F*^ mice and a clear differentiation from WT mice. Overall, stronger affinity to TfR is a critical feature when designing future bispecific immunoPET radioligands for CNS targets that will leverage TfR-mediated transcytosis for crossing the BBB.

## Electronic supplementary material

Below is the link to the electronic supplementary material.

Supplementary Material 1

## Data Availability

The datasets generated and/or analysed during the current study are available from the corresponding author on reasonable request.

## Abbreviations

[^11^C]PiB: [^11^C]Pittsburgh Compound B
^124^I: iodine-124
^125^I: iodine-125
Aβ: amyloid-beta
AD: Alzheimer’s disease
AUC: area under the curve
AUC_blood_: AUC for the blood pharmacokinetics
AUC_brain_: AUC for the brain pharmacokinetics
AUC_brain_/AUC_blood_: ratio of AUC_brain_ and AUC_blood_
Bapi: bapineuzumab
BBB: blood-brain barrier
CNS: central nervous system
HRP: horseradish peroxidase
KiH: knob-into-hole
MFI: mean fluorescent intensity
mTfR: mouse transferrin receptor 1
OD: optical density
PBS: phosphate buffered saline
PET: positron emission tomography: TfR: transferrin receptor
WT: wild-type
