# Supplementary material for: High-affinity transferrin receptor binding improves brain delivery of bispecific antibodies at tracer dose
Source: Fluids Barriers CNS. 2025 Aug 21;22:86. doi: 10.1186/s12987-025-00693-2 (PMC12369151; doi:10.1186/s12987-025-00693-2)
Supplement: Supplementary file 1 — Supplementary Material 1 [file 12987_2025_693_MOESM1_ESM.docx]

## Supplementary Tables

Table S1. Number of mice (male/female), injected dose and activity for each antibody.

| **Radioligand** |  | **4 h** | | **24 h** | | **72 h** | | **7 d** | |  | **Injected dose**  **(mg/kg)** | **Injected activity**  **(MBq)** |
| --- | --- | --- | --- | --- | --- | --- | --- | --- | --- | --- | --- | --- |
|  |  | **WT** | ***App^NL-G-F^*** | **WT** | ***App^NL-G-F^*** | **WT** | ***App^NL-G-F^*** | **WT** | ***App^NL-G-F^*** |  |  |  |
| [^125^I]I-Bapi-8D3_WT_ |  | (2,1) | (2,1) | (1,2) | (2,1) | (1,2) | (2,1) | (1,2) | (2,1) |  | 0.22 ± 0.03 | 0.96 ± 0.24 |
| [^125^I]I-Bapi-8D3_Y32A_ |  | (1,2) | (2,1) | (1,2) | (1,2) | (1,2) | (1,2) | (1,2) | (2,1) |  | 0.19 ± 0.02 | 0.98 ± 0.20 |
| [^125^I]I-Bapi-8D3_Y52A_ |  | (1,2) | (2,1) | (2,2) | (2,1) | (1,2) | (1,3) | (1,2) | (2,1) |  | 0.21 ± 0.02 | 1.00 ± 0.22 |
| [^125^I]I‑Bapi |  | (1,2) | (2,1) | (1,2) | (1,2) | (2,1) | (1,2) | (1,2) | (2,1) |  | 0.20 ± 0.01 | 1.04 ± 0.15 |
| [^124^I]I-Bapi-8D3_WT_ |  |  |  |  |  | (2,2) | (2,2) |  |  |  | 1.15 ± 0.01 | 4.19 ± 0.50 |

Data presented as mean ± standard deviation.

Table S2. EC50 values with 95% confidence intervals for Bapi-8D3_WT_, Bapi-8D3_Y32A_ and Bapi-8D3_Y52A_ binding to mTfR in the on-cell affinity assay.

| **Antibody** | **EC50 (nM)** | **95% confidence interval** |
| --- | --- | --- |
| Bapi-8D3_WT_ | 10.0 | 8.1-12 |
| Bapi-8D3_Y32A_ | 19.6 | 16-24 |
| Bapi-8D3_Y52A_ | 241 | *Indeterminable* |

## Supplementary Figures


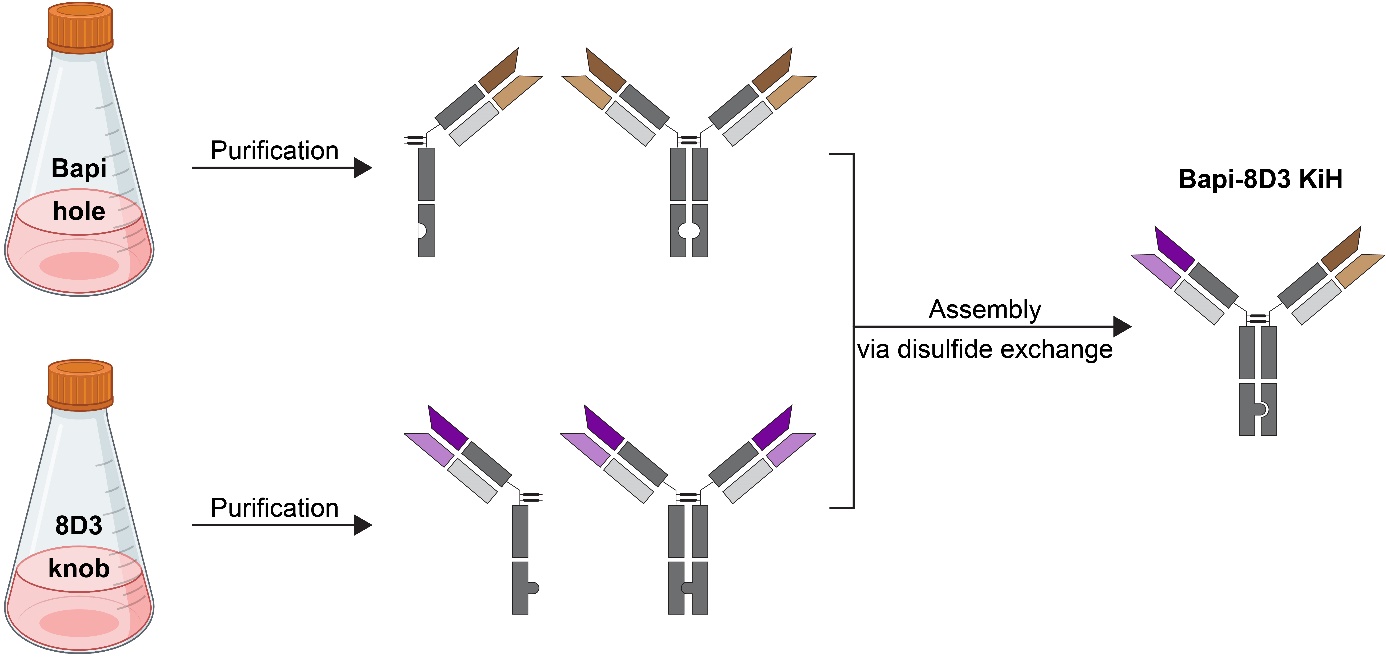


**Figure S1.** Schematic illustrating production of KiH bispecific antibodies. Knob and hole antibodies were produced separately by ExpiCHO cells (left). Following purification, knob and hole antibodies were assembled via disulphide exchange to produce bispecific KiH antibodies.


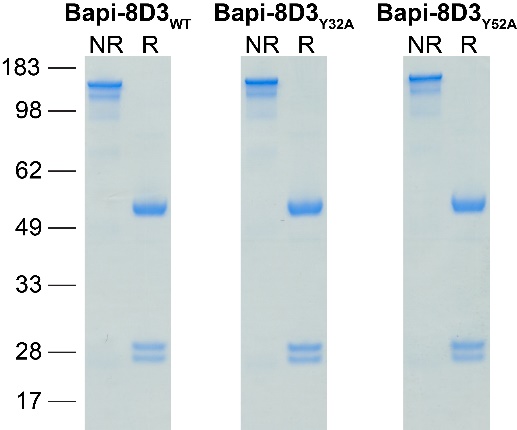


**Figure S2.** SDS-PAGE analysis of Bapi-8D3_WT_, Bapi-8D3_Y32A_, Bapi-8D3_Y52A_ in non-reducing (NR) and reducing (R) conditions.


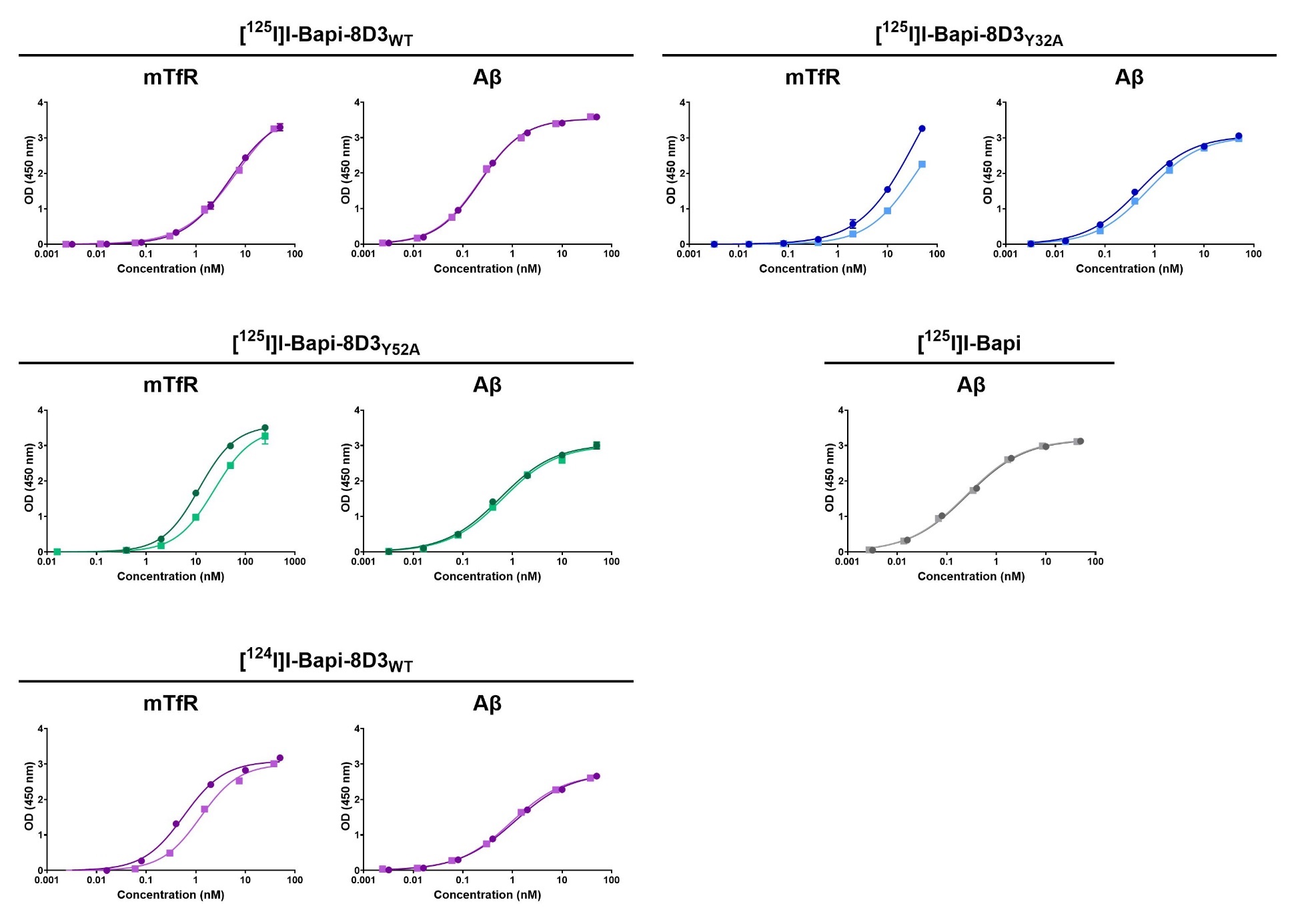


**Figure S3.** ELISA analysis of antibodies binding to mTfR and Aβ before (circle) and after (square) radiolabelling.


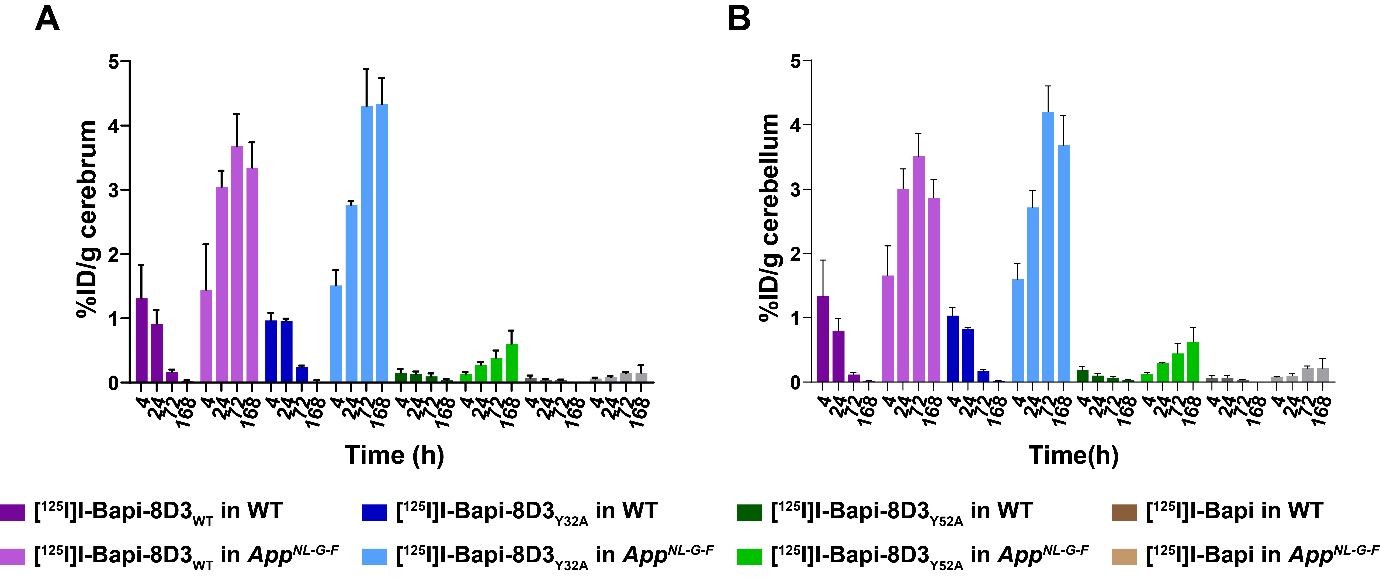


**Figure S4.** *Ex vivo* cerebral (**A**) and cerebellar (**B**) concentrations (%ID/g) of [^125^I]I-Bapi-8D3_WT_, [^125^I]I-Bapi-8D3_Y32A_, [^125^I]I-Bapi-8D3_Y52A_ and [^125^I]I-Bapi in WT and *App^NL-G-F^* mice over 168 h post-injection.


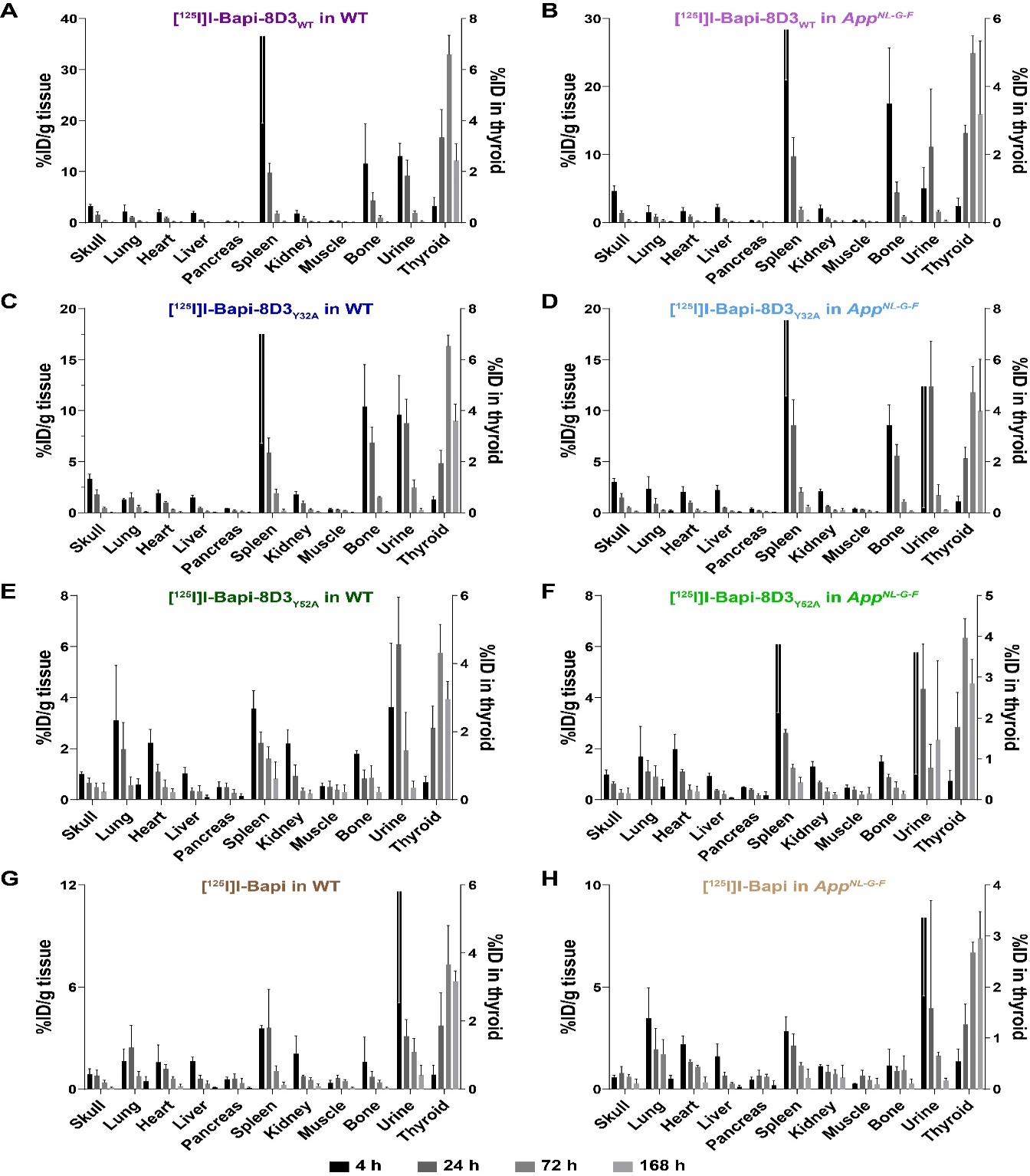


**Figure S5.** Peripheral *ex vivo* biodistribution (%ID/g organ/urine or %ID in thyroid) of [^125^I]I-Bapi-8D3_WT_ (**A, B**), [^125^I]I-Bapi-8D3_Y32A_ (**C, D**), [^125^I]I-Bapi-8D3_Y52A_ (**E, F**) or [^125^I]I-Bapi (**G, H**) in WT (**A, C, E, G**) and *App^NL-G-F^* (**B, D, F, H**) mice 4, 24, 72 and 168 h post-injection.
